# Supplementary material for: Intraperitoneal delivery of the oncolytic virus CF17 improves tumor immunogenicity and survival in gastric cancer peritoneal metastasis
Source: Mol Ther Oncol. 2026 Jun 13;34(3):201266. doi: 10.1016/j.omton.2026.201266 (PMC13333358; doi:10.1016/j.omton.2026.201266)
Supplement: Document S1. Figures S1–S2 [file mmc1.pdf]

OMTON, Volume *34*

Supplemental information

Intraperitoneal delivery of the oncolytic virus CF17 improves tumor immunogenicity and survival in gastric cancer peritoneal metastasis

Zhifang Zhang, Annie Yang, Anthony K. Park, Shyambabu Chaurasiya, Sang-In Kim, Jianming Lu, Isabel Monroy, Hannah Valencia, Courtney Chen, Supriya Deshpande, Yuman Fong, and Yanghee Woo

Figure S1

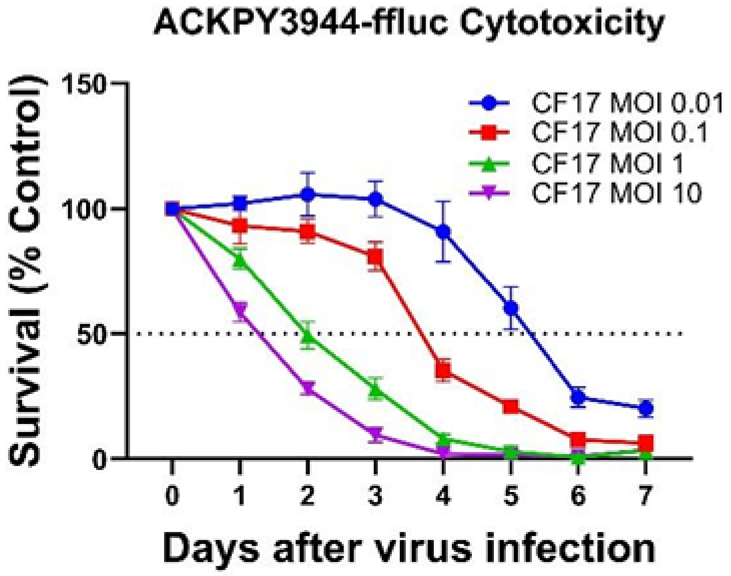

Figure S1. Cytotoxicity of CF17 in ACKPY3944-ffluc cells is similar to that of parental ACKPY3944 cells, related to Figure 2. ACKPY3944 cells were engineered to stably express firefly luciferase (ffluc) via lentiviral transduction and infected with CF17 at the indicated multiplicities of infection (MOIs). Cell survival, relative to mock-infected controls, was measured daily for 7 days post-infection. All experiments were performed thrice, and data are presented as mean ± SEM.

Figure S2

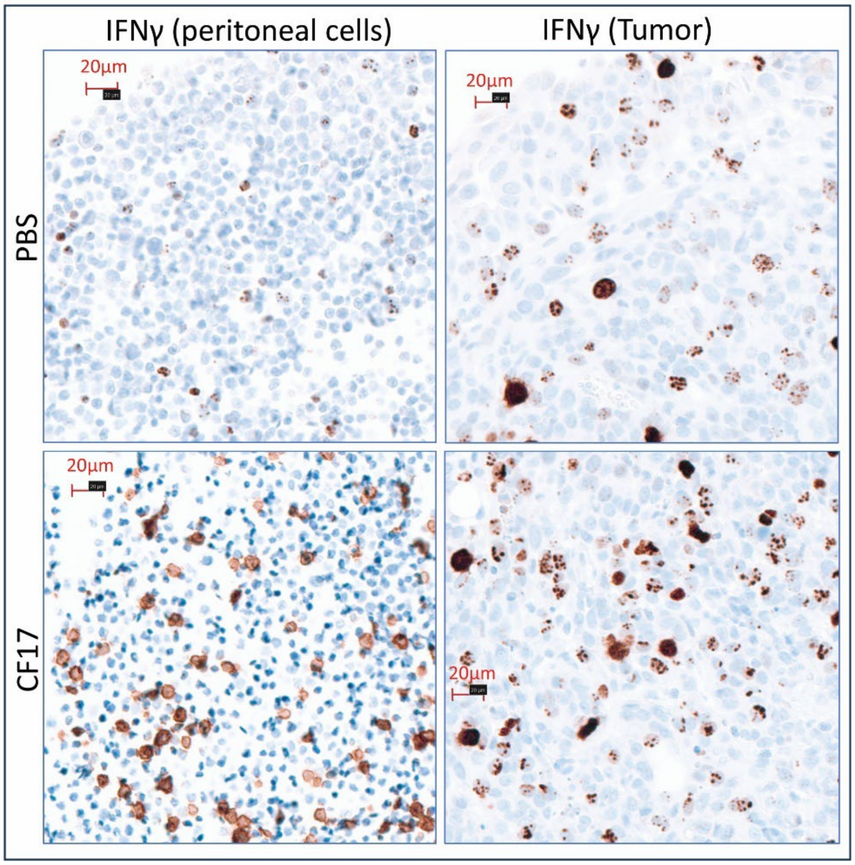

Figure S2. CF17 treatment increases IFNγ⁺ cells in peritoneal cells and tumors after 9 days of treatment, related to Figure 6. Representative immunohistochemistry staining for IFNγ in peritoneal cells (left) and tumor sections (right) from mice treated intraperitoneally with PBS (top) or CF17 (bottom) for 9 days. Scale bar = 20 μm.
