# Supplementary material for: Intraperitoneal delivery of the oncolytic virus CF17 improves tumor immunogenicity and survival in gastric cancer peritoneal metastasis
Source: Mol Ther Oncol. 2026 Jun 13;34(3):201266. doi: 10.1016/j.omton.2026.201266 (PMC13333358; doi:10.1016/j.omton.2026.201266)
Supplement: Document S2. Article plus supplemental information [file mmc2.pdf]

Molecular Therapy
Oncology
Original Article

Intraperitoneal delivery of the oncolytic virus CF17 improves tumor immunogenicity and survival in gastric cancer peritoneal metastasis

Zhifang Zhang,[1,3] Annie Yang,[1,3] Anthony K. Park,[2] Shyambabu Chaurasiya,[1] Sang-In Kim,[1] Jianming Lu,[1] Isabel Monroy,[2] Hannah Valencia,[1] Courtney Chen,[1] Supriya Deshpande,[1] Yuman Fong,[1] and Yanghee Woo[1,2]

[1]Division of Surgical Oncology, Department of Surgery, City of Hope National Medical Center, 1500 E Duarte Road, Duarte, CA 91010, USA; [2]Cancer Immunotherapeutics Program, Beckman Research Institute, City of Hope National Medical Center, 1500 E Duarte Road, Duarte, CA 91010, USA

Peritoneal metastasis (PM) is the leading cause of treatment failure and death from gastric cancer (GC). We evaluated the antitumor and immunogenic effects of CF17, an oncolytic chimeric orthopoxvirus, in a novel syngeneic mouse model of GCPM. CF17 was tested in mouse GC cell lines (ACKPY3944 and ACKPY4113) for viral replication and cytotoxicity. A syngeneic mouse model of PM was established in C57BL/6 mice via intraperitoneal (IP) implantation of 1×10^6 ACKPY3944-ffluc cells. Mice were treated with either IP CF17 or PBS. Peritoneal tumor burden was monitored by bioluminescence imaging. Immune profiles of peritoneal washings and tumors were assessed by flow cytometry and immunohistochemistry. CF17 infected, replicated in, and killed ACKPY3944 and ACKPY4113 cells *in vitro*. Mice treated with IP CF17 showed a significant decrease in tumor burden ($p < 0.05$) and prolonged survival ($p < 0.001$). CF17 treatment significantly enhanced leukocyte infiltration, including $CD4^+$ and $CD8^+$ cells, into the tumor microenvironment of the peritoneal cavities and solid tumors. Overall, these results demonstrate that CF17 has potent anti-tumor activity and immunogenicity in a syngeneic mouse model of GCPM. These data provide a framework to support future studies to optimize CF17-based immunotherapeutic approaches for GCPM.

INTRODUCTION

Gastric cancer (GC) remains a major global health burden being the fifth most commonly diagnosed cancer and the third leading cause of cancer-related deaths.[1,2] At initial diagnosis, 15%–30% of the patients present with peritoneal metastasis (PM),[3,4] and an additional 40%–60% develop peritoneal recurrence after curative-intent surgery and systemic chemotherapy.[4,5] Despite modest improvements with PD-L1, HER2, CLND18.2, or mismatch repair deficiency targeting therapies, the prognosis for patients with peritoneal metastasis from gastric cancer (GCPM) is less than 8 months.[6] Peritoneal-directed therapy such as cytoreductive surgery (CRS) combined with hyperthermic intraperitoneal chemotherapy (HIPEC),[6,7] catheter-based intraperitoneal chemotherapy, and pressurized intraperitoneal aerosol chemotherapy (PIPAC) offer limited benefit in select patients and are associated with substantial morbidity.[8,9] These limitations highlight the urgent need for novel, effective, and well-tolerated therapeutic strategies for GCPM.

Oncolytic virotherapy is a promising cancer treatment strategy that harnesses the innate and genetically engineered properties of viruses to selectively infect, replicate within, and lyse cancer cells, while simultaneously stimulating immune responses and exposing tumor antigens to the immune system.[10] Oncolytic viruses (OVs) can be armed to deliver immunomodulatory transgenes, thereby reshaping the tumor microenvironment (TME) and enhancing systemic immunity.[11] These dual mechanisms of direct tumor destruction and immune activation make OVs an especially attractive therapy for immunogenically “cold” solid tumors with limited therapeutic options, including PM from diffuse-type GC.

While many OVs have entered clinical trials, most have demonstrated limited success in solid tumors, particularly intra-abdominal cancers, where intravenous delivery is hindered by systemic clearance, immune neutralization, and poor biodistribution to peritoneal surfaces. Direct intratumoral (IT) injections, often used for accessible solid tumors, are not feasible for PM. Therefore, effective regional intraperitoneal (IP) delivery of potent and tumor-selective OVs may offer a superior therapeutic strategy in GCPM.

CF17 is a novel oncolytic chimeric orthopoxvirus developed through random recombination of nine orthopoxvirus species. CF17 was

Received 23 September 2025; accepted 11 June 2026;
https://doi.org/10.1016/j.omton.2026.201266.

[3]These authors contributed equally

Correspondence: Zhifang Zhang, Assistant Research Professor, Department of Surgery, City of Hope National Medical Center, 1500 E Duarte Road, Duarte, CA 91010, USA.
E-mail: zhzhang@coh.org
Correspondence: Yanghee Woo, Division of Surgical Oncology, Department of Surgery, City of Hope National Medical Center 1500 E. Duarte Road, Duarte, CA 91010, USA.
E-mail: yhwoo@coh.org

Published by Elsevier Inc. on behalf of The American Society of Gene and Cell Therapy.
This is an open access article under the CC BY license (http://creativecommons.org/licenses/by/4.0/).

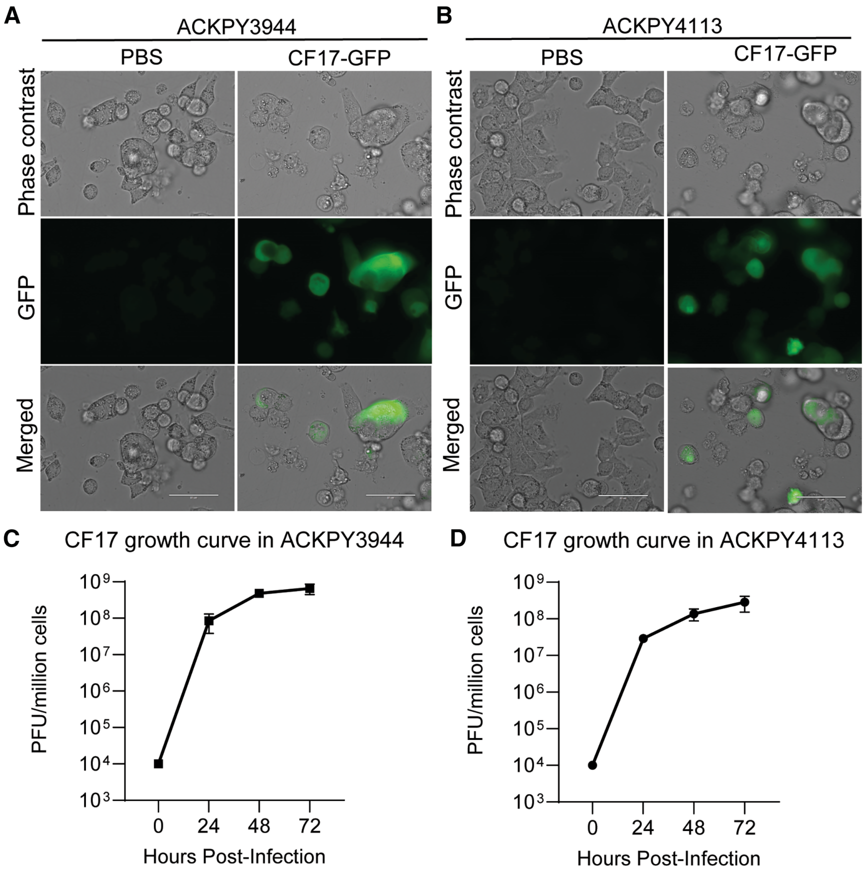

Figure 1. CF17 viruses replicate in mouse gastric cancer cell lines ACKPY3944 and ACKPY4113

(A and B) Mouse GC cells ACKPY3944 (A) and ACKPY4113 (B) were either mock-infected (PBS, *n* = 3) or infected with CF17-GFP (MOI = 3, *n* = 3) and imaged for GFP 18-h post-infection. (C and D) Mouse GC cell lines ACKPY 3944 (C) and ACKPY4113 (D) were infected with CF17 (MOI = 0.01, *n* = 3 each) and virus titer in the cell lysate was determined at indicated time points using a standard plaque assay. All experiments were performed in triplicates. Data presented as mean ± SEM.

selected among 100 different chimeric candidates based on its potent oncolytic activity in an NCI-60 cell panel[12,13] and in human GC lines, including both intestinal type and diffuse type.[14] Previously, we demonstrated that CF17 treatment prolongs survival in a xenograft mouse model of the human SNU-16 cell line.[14] In this study, we sought to evaluate the efficacy of IP delivered CF17 in a novel syngeneic immunocompetent mouse model of GCPM using a mouse ACKPY3944-ffluc cell line.

RESULTS

CF17 replicates in mouse GC cells

Since CF17 was screened against a human NCI-60 cancer cell panel, we investigated whether CF17 can infect and replicate in the mouse GC cell lines, ACKPY3944 and ACKPY4113.[15] We used the CF17-GFP virus (multiplicity of infection: MOI = 3) to infect these cells and determined virus replication indirectly by monitoring GFP levels and directly by standard plaque assay. GFP signal was clearly observed in both cell lines after 18 h post-infection (Figures 1A and 1B). Then, we quantified the replication of CF17 in both cell lines. Cells were collected daily for three days following treatment with CF17 (MOI = 0.01). The virus growth kinetics in cells was determined using a standard plaque assay. Our results showed that virus titers reached a plateau at 48 or 72 h after infection and increased by more than 4-log scales compared to the input virus (0-h time point) in both ACKPY3944 and ACKPY4113 cell lines (Figures 1C and 1D). These results indicate that CF17 infects and robustly replicates in mouse GC cells.

CF17 efficiently kills mouse GC cells

Next, we verified the cytotoxic properties of CF17 in the ACKPY3944 and ACKPY4113 cell lines. Cells were treated with CF17 at four different MOIs (0.01, 0.1, 1, or 10) and cell survival was determined over 8 days. Our results showed a dose- and time-dependent killing in both cell lines (Figure 2). Even at the lowest MOI tested (0.01), CF17 showed significant cytotoxicity in both cell lines. More than 95% ACKPY3944 and ACKPY4113 cells were killed at day 7 with an MOI = 0.1, at day 5 with an MOI = 1, and at day 3 with an MOI = 10. There was no difference in cell killing between two cell lines. These results suggest that CF17 effectively kills mouse GC cell lines ACKPY3944 and ACKPY4113 in a dose- and time-dependent manner.

A novel syngeneic mouse model of GCPM with ACKPY3944-ffluc cells

Next, we tested the efficacy of CF17 *in vivo* and assessed how it affects TME. We developed a novel syngeneic model of GCPM using ACKPY3944-ffluc cells. Since the ACKPY3944 cells are derived from primary tumor, while ACKPY4113 cells are from tumors in lymph node metastasis,[15] we chose the ACKPY3944 cell line to evaluate its ability to form tumors in the peritoneal cavity of C57BL/6 mice. Cytotoxicity assays for ACKPY3944-ffluc cells treated with CF17 (Figure S1), were identical to those for ACKPY3944 cells treated with CF17 (Figure 2), suggesting that luciferase transfection did not alter the cytotoxicity of CF17. 1×10^6 ACKPY3944-ffluc cells were implanted at D0 by IP injection and bioluminescence imaging was performed weekly to check the tumor burden (Figure 3A). After IP implantation of ACKPY3944-ffluc, tumor burden changes were examined over time (Figure 3B). Our results showed that IP implantation of 1×10^6 cells led to development of GCPM. One representative mouse, harvested on day 9 post-implantation, demonstrated

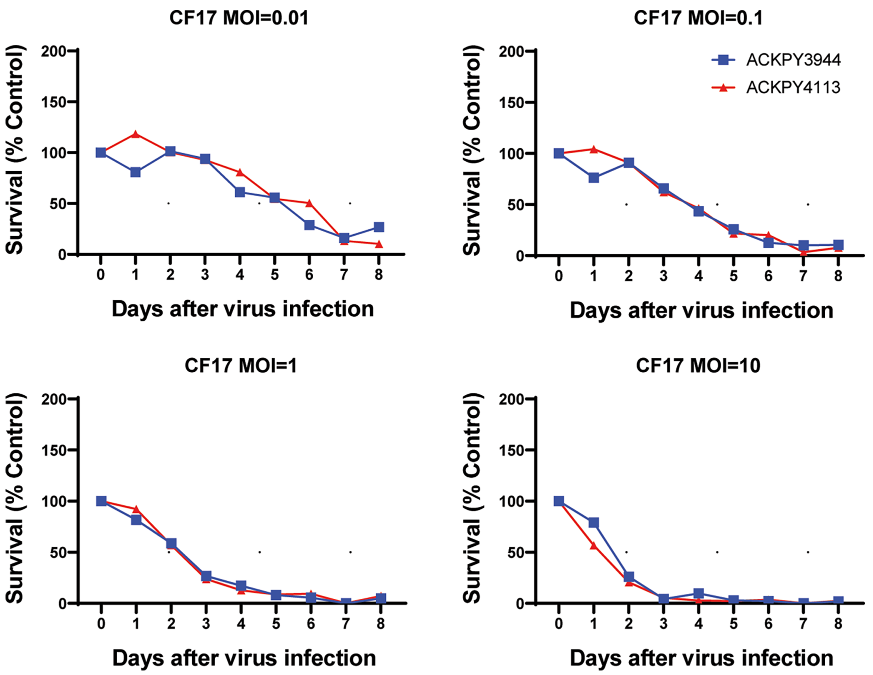

Figure 2. Cytotoxicity of CF17 in mouse gastric cancer cell lines ACKPY3944 and ACKPY4113

Mouse GC cell lines ACKPY3944 and ACKPY4113 were infected with CF17 at indicated MOIs. Cell survival relative to mock-infected cells was calculated daily post-infection for 8 days. All experiments were performed at least three times. Data are shown as mean ± SEM.

peritoneal dissemination of tumor (Figure 3C). Total tumor burden changes (Figure 3D) with individual tumor burden changes (Figure 3E) are shown as Photons/sec/ROI. Results showed that IP implantation of 1×10^6 ACKPY3944-ffluc cells successfully established an immunocompetent model of GCPM with stable tumor growth in C57BL6 mice.

IP CF17 treatment reduces tumor burden in the syngeneic ACKPY3944-ffluc-GCPM mouse model

The experimental design of IP CF17 treatment is shown in Figure 4A. On day 3 post-IP implantation of ACKYP3944-ffluc cells, mice were divided into two groups based on average tumor burden. One group received IP PBS control treatment (n = 8) and the second group received IP CF17 treatment (n = 8). Peritoneal tumor burden changes were observed using bioluminescence imaging system (Figure 4B). Peritoneal tumor burden significantly decreased in the CF17-treated group compared to the PBS control group on day 7 ($p < 0.05$) (Figure 4C). Individual tumor burden changes of two groups are shown in Figure 4D. Our results show that IP CF17 treatment significantly decreases tumor burden in a syngeneic ACKPY3944-ffluc-GCPM mouse model.

IP CF17 treatment prolongs survival and changes the TME in a syngeneic ACKPY3944-ffluc-GCPM mouse model

To further observe the efficacy of CF17 treatment on mouse survival we performed a Kaplan-Meier survival analysis. Results showed that the last surviving mouse in IP PBS treatment group died on D33, whereas six mice survived in the IP CF17-treated group. Body weight changes are shown in Figure 5A. Also, CF17 treatment significantly increased overall survival ($p < 0.01$) (Figure 5B). To evaluate the TME change of GCPM post IP CF17 treatment, both peritoneal lavages and tumors were harvested on day 9 post ACKPY3944-ffluc implantation (also on day 2 after third IP CF17 or PBS treatment). The total cell numbers in peritoneal lavage of IP CF17-treated group were significantly higher than in the IP PBS-treated group ($p < 0.05$, Figure 5C). We included leukocyte surface markers CD45, CD3, CD8, and CD4 in the flow cytometric analysis to separate subsets of peritoneal cells. Our results showed that there is no significant change of cancer cell number ($CD45^-$/larger size cells)[16] between the two groups due to larger deviation in the PBS group; although cancer cell numbers in the CF17-treated group was much lower than in the PBS-treated group. $CD45^+$ leukocytes were the most enriched population in the peritoneal lavage ($p < 0.05$). In the subsets of leukocytes, IP CF17 treatment significantly increased $CD3^+$ T cells (5 times) and $CD8^+$ T cells (10 times) compared to PBS treatment ($p < 0.05$ or $p < 0.01$, respectively). There was no significant difference in the number of $CD4^+$ T cells between the two groups. Peritoneal cells were also stained using immunohistochemistry (IHC) for H&E, CD8, and CD4 antibodies to visually observe the change of $CD8^+$ T cells and $CD4^+$ T cells. The results of IHC were in accordance with the results of flow cytometric analysis (Figure 6A). i.e., increase of $CD8^+$ T cells in peritoneal cavities.

Furthermore, peritoneal tumors were collected and stained with H&E, CD8, and CD4 to evaluate immune TME. Our results showed an increase in $CD8^+$ T cells in both the solid tumors and peritoneal cells of IP CF17-treated group (Figure 6B). Notably, $CD4^+$ T cells also increased in the tumors of the IP CF17-treated group compared to the PBS-treated group, while such an increase was not seen in peritoneal $CD4^+$ T cells. Additionally, we observed increased IHC staining for $IFN\gamma^+$ cells in the peritoneal cavity and solid tumor post-virus treatment (Figure S2). Overall, these results indicate that IP CF17 treatment significantly prolongs survival and changes the TME of GCPM of both peritoneal cavities and tumors by increasing the number of $CD8^+$ T cells.

DISCUSSION

Oncolytic virotherapy offers a dual mechanism for cancer cell death by direct tumor lysis along with an immune activation response. We previously reported that CF17 demonstrated potent

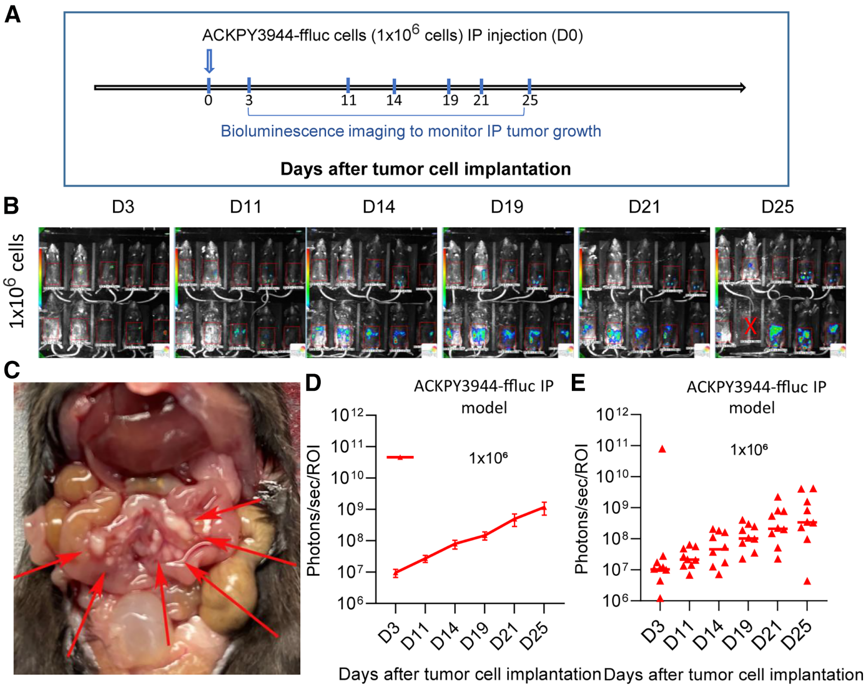

Figure 3. Syngeneic ACKPY3944-ffluc-GCPM mouse model

(A) The timeline shows intraperitoneal (IP) implantation of ACKPY3944-ffluc cells and bioluminescence imaging in C57BL/6 mice (n = 10, including 5 female [upper row] and 5 male mice [lower row]). (B) Bioluminescence imaging of the region of interest (ROI) of IP tumor burden. (C–E) Formation of peritoneal tumor at day 9 post-IP injection of ACKPY3944-ffluc (red arrows show the tumor). Summarized tumor burdens (D) and individual tumor burdens (E) post-IP injection of ACKPY3944-ffluc. Mean ± SEM.

cytotoxicity against human GC cell lines and offered a survival benefit in an immunodeficient xenograft mouse model. However, immune-mediated anti-tumor effects of OVs cannot be evaluated in immunocompromised models. To better mimic the human TME, we established a syngeneic, immunocompetent mouse model of GCPM using ACKPY3944-ffluc cells. In this model, CF17 exhibited strong infectivity and cytotoxicity against both ACKPY3944 and ACKPY4113 mouse GC cell lines. IP administration of CF17 significantly reduced tumor burden and prolonged survival. Importantly, CF17 treatment increased $CD8^+$ T cell infiltration within the peritoneal cavity and tumors, suggesting that CF17 reprograms the local immune microenvironment and triggers an antitumor immune response.

Since this was a preliminary investigation, one limitation of our analysis on day 9 was a small sample size (n = 3), hence an unpaired t test (assuming a normal Gaussian distribution) showed a significant difference between the CF17-treated group and PBS control group, whereas this difference was not significant with the nonparametric Mann-Whitney test. Future studies will include power and sample size calculations. However, given the scarcity of robust immunocompetent GCPM models, the successful development of an IP ACKPY3944-ffluc-GCPM mouse model provides a valuable platform for evaluating novel therapies for treating GCPM. Although other murine models (e.g., YTN16, T3-2D) have been described, they are either not widely available or have not been validated in the peritoneal setting.[17,18] The peritoneal cavity is lined by a thin layer of the peritoneum, and the peritoneal niche is often considered a "soil" where certain epithelial malignancies are characteristically "seeded".[19,20] Our results showed that IP implantation of ACKPY3944-ffluc induces peritoneal dissemination with linear growth of tumor burden. By using this novel mouse model, we demonstrated the oncolytic function of CF17 with $CD8^+$ T cells increasing in TME of both peritoneal cavities and solid tumors.

To date, numerous OVs—both naturally occurring (e.g., reovirus, VSV) and genetically modified (e.g., adenovirus, HSV, vaccinia)—have shown promise in preclinical studies.[21,22]

CF17-GFP, a ∼190 kb chimeric poxvirus with a GFP insertion at the thymidine kinase (TK) locus, is engineered for tracking and future genetic modification. Strategic deletions (e.g., TK, B18R, VGF) in vaccinia viruses have been shown to enhance tumor selectivity, modulate immune evasion, and improve safety. Although we did not include detailed safety and biodistribution analyses in the current study, we will address these in future studies to enhance the translational context and provide a more balanced interpretation. Further optimization of CF17 through incorporation of immune-stimulating payloads (e.g., cytokines or chemokines) could augment its therapeutic efficacy.[23–27] Several prior studies have shown that arming OVs with CCL5, CXCL11, or other immune effectors improves T cell and NK cell recruitment, enhances tumor suppression, and potentiates systemic immune responses.[28] For example, CCL5-armed oncolytic VACV WR (vvCCL5) increased TILs and exerted a significant tumor suppressive effect when used to simultaneously vaccinate its receptor type-1-polarized dendritic cells.[29] CCL5-expressing oncolytic VACV WR (OVffLuc-CCL5) improved NK cell infiltration within tumors *in vivo*.[30] Oncolytic VACV WR (vvDD) armed with CXCL11 (vvDD-CXCL11) recruited $CD8^+$ T cells and, to a lesser extent, NK cells to the TME to trigger a systemic antitumor immune response.[31] Moon et al. demonstrated that vvDD-CXCL11 successfully recruited T cells and augmented antitumor efficacy.[32] IP immunotherapy with an oncolytic VACV WR (mJX-594) in a peritoneal carcinomatosis model of colon cancer, with or without anti-PD-1 antibody, reduced peritoneal cancer progression and formation of malignant ascites. This study showed IT activation of dendritic cells and restored effector

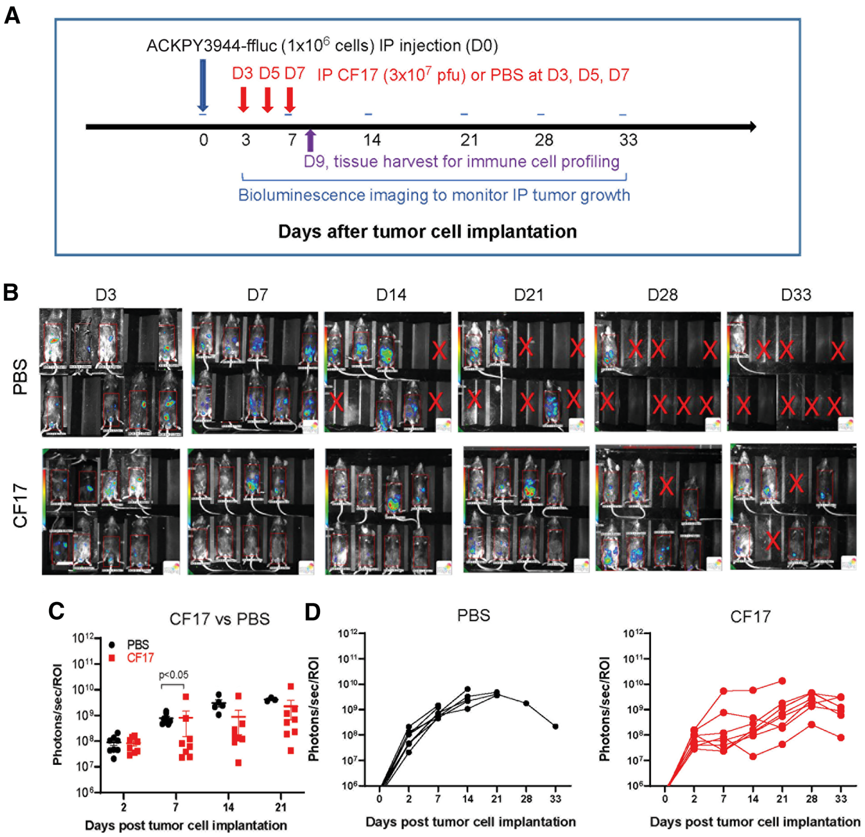

Figure 4. Timeline and IP CF17 treatment in syngeneic ACKPY3944-ffluc-GCPM mouse model

(A) The timeline shows intraperitoneal implantation of 1×10^6 ACKPY3944-ffluc cells in C57BL/6 mice, IP treatment with PBS (n = 8) or CF17 (n = 8), and bioluminescence imaging time points. C57BL/6 mice were IP injected with PBS or CF17 3 times (on day 3, day 5, and day 7 post-cancer cells implantation at a dose of 3×10^7 pfu in 400 μL PBS). (B) Bioluminescence imaging of the region of interest (ROI) of IP tumor burden. (C) Statistical analysis of tumor burden after treatment with IP PBS or IP CF17 and (D) individual tumor burden change. Mean ± SEM.

functions of CD8$^+$ T cells in the peritoneal cavity leading to anticancer immunity within the TME, which was stronger when combined with immune checkpoint blockade.[33] In another study, a modified VACV WR Ankara (MVA) expressing single-cell interleukin 12 (scIL-12) was shown to completely eradicate MC38 colon cancer cells implanted in the peritoneal cavity and spleen of mice by inducing tumor-specific CD8$^+$ T lymphocytes and the development of systemic immune response. Furthermore, cured mice showed protection following re-challenge experiments.[34] A recombinant VACV WR strain expressing mutant survivin (SurT34A) and FilC was shown to replicate in GC cells and to be safe at high doses *in vivo*. Adaptive immune responses that included upregulation of CD4$^+$ and CD8$^+$ T cells were shown to play an important role in tumor regression.[35]

Several OVs have been clinically approved in the past two decades to establish OVs as safe and viable anticancer agents. In 2005, a genetically modified adenovirus H101 was approved in China for the treatment of head and neck cancer. A decade later in 2015 herpesvirus talimogene laherparepvec (OncoVex, T-VEC) was approved in the U.S. for melanoma, while a genetically engineered herpes simplex virus type 1, G47Δ (Teserpaturev, Delytact) was approved in Japan in 2021 for malignant glioma. Furthermore, a non-replicating adenoviral vector-based gene therapy was approved in the U.S. in 2022 for the treatment of bladder cancer.[36,37] Additionally, a clinical trial involving IT injections of a VACV WR (VV), engineered for tumor selectivity through two targeted gene deletions (vvDD), in patients with advanced solid tumors demonstrated tumor specificity and antitumor activity, and was well tolerated in these patients.[38] IP oncolytic viral therapy with a modified VACV WR, Olvi-Vec, was also shown to be clinically safe and resulted in immune activation in patients with platinum-resistant or refractory ovarian cancer.[39]

Given the complexity of OV activity within the TME, combination strategies with checkpoint inhibitors, cytokine therapies, or conventional treatments may be more effective than monotherapy.[10] Our findings lay the foundation for rational design of enhanced CF17 constructs optimized for immunogenicity and tumor selectivity.

In summary, CF17 effectively kills murine GC cell lines *in vitro* and demonstrates robust anti-tumor activity and survival benefit in a novel syngeneic mouse model of GCPM *in vivo*. CF17 shows significant antitumor activity and survival benefit *in vivo*. Its ability to enhance CD8$^+$ T cell infiltration suggests immune activation within the peritoneal TME. These data support further development of CF17 as a regionally delivered oncolytic immunotherapy for GCPM patients.

MATERIALS AND METHODS

Generation of CF17 and CF17-GFP

CF17 is a chimeric poxvirus generated from nine strains of orthopoxvirus.[12–14] Briefly, all nine strains of orthopoxvirus were used to co-infect CV-1 cells (ATCC, catalog# CCL-70) to foster chimerization. Then, viral plaques were chosen and purified through three rounds of plaque purification to obtain 100 unique clonally purified chimeric orthopoxviruses. High-throughput screening was used to compare cytotoxic efficacy against the NCI-60 panel. CF17 was selected as a chimeric isolate with its superior cell killing in the NCI-60 panel when compared with all parental viruses. CF17-GFP was generated by inserting a cDNA encoding Emerald (GFP) under the control of the H5 early/late promoter into the CF17 genome by replacing the viral J2R locus via homologous recombination. Briefly,

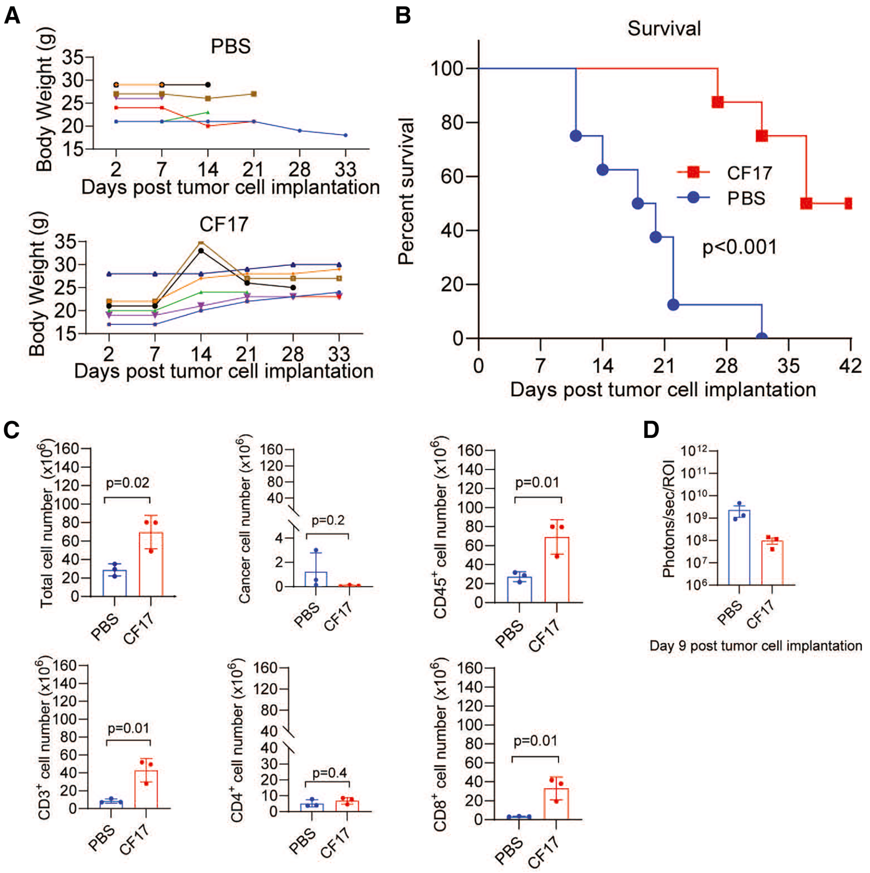

Figure 5. IP CF17 treatment significantly prolongs survival and increases CD8+ T cells in the microenvironment of peritoneal cavity

(A) Body weight change of mice treated with CF17 or PBS 3 times (on day 3, day 5, and day 7 after tumor cell implantation at a dose of 3×10^7 pfu in 400 μL PBS) over time. (B) Kaplan-Meier survival analysis of IP ACKPY3944 tumor-bearing mice following treatment with IP CF17 (n = 8) or PBS control (n = 8). (C) On day 9 post-IP ACKPY3944-ffluc implantation (i.e., 2 days after third injection of CF17), peritoneal lavage was collected by washing with 5 mL PBS; cells were counted by using hemocytometer, stained with antibodies shown in the figure, and analyzed using flow cytometric analysis (n = 3 each group). Cell numbers were calculated with total cell number (hemocytometer) x percentage of each marker of subsets (flow cytometric analysis). (D) Bioluminescence imaging on day 9 post-IP ACKPY3944-ffluc implantation. Mean ± SEM.

the GFP expression cassette driven by the VACV H5 promoter was PCR-amplified from the plasmid Emerald-pBAD (Addgene, Cambridge, MA). The resulting PCR product was digested with SacI and BamHI and cloned into the shuttle plasmid pNC-TK to generate pNC-TK-GFP in which the GFP expression cassette is flanked by sequences from left and right flank of viral J2R gene. Next, CV-1 cells were infected with the "wild-type" CF17 at an MOI 0.1 followed by transfection with the shuttle plasmid (pNC-TK-GFP). After 48 h of incubation, cell lysates were harvested and used for further infection of CV-1 cells. Recombinant virus (CF17-GFP) plaque was identified and selected under fluorescent microscope and was further purified by several rounds of plaque purification.[40]

Cell lines and culture

Transgenic mouse GC cell lines ACKPY3944 (from primary tumor) and ACKPY4113 (tumor cells from lymph node metastasis) were generated by Dr. Sandra Ryeom's lab (University of Pennsylvania) from Atp4b-Cre; Cdh1fl/fl; LSL-KrasG12D; Trp53fl/fl; Rosa26LSL-YFP (ACKPY) mice (C57BL6 background),[15] on the basis of conditional deletion of E-cadherin(*Cdh1*) and p53 (*Trp53*) driven by ATP4B-cre, expressed exclusively in the gastric parietal cell lineage.[41] African green monkey kidney fibroblast CV-1 (ATCC, catalog# CCL-70) were purchased from the American Type Culture Collection (ATCC, Manassas, VA). ACKPY3944, ACKPY4113, and CV-1 were cultured in DMEM (Catalog# 10-017-CV) supplemented with 10% fetal bovine serum (FBS, catalog# 35-010-CV) and 1% antibiotic-antimycotic solution (AAS, catalog# 30-004-Cl). All the media and supplements were purchased from Corning (Corning, NY). The cells were maintained in a humidified incubator at 37°C and 5% CO_2. For all adherent cell lines, when adherent cells reached 80% confluency, they were passaged using 0.05% trypsin and EDTA solution (Corning, catalog# 25-051-Cl). Media were changed every 2–3 days.

GFP fluorescent imaging *in vitro*

ACKPY3944 and ACKPY4113 cells ($1x10^5$/well) were plated in 24-well plates and incubated for 24 h before infected with PBS or CF17-GFP at multiplicity of infection (MOI) = 3. This means that for every cell, there are 3 plaque-forming units (PFU) of the virus added, resulting in a cells-to-virus ratio of 1:3. After infection for 18 h, cells were imaged for virus-encoded GFP using an EVOS FL Auto Cell Imaging System (Life Technologies Corporation, Carlsbad, CA, USA). All images were adjusted identically.

Virus infection and proliferation assay

ACKPY3944 and ACKPY4113 cells were seeded in 6-well plates at a density of 5×10^5 cells per well and allowed to adhere overnight. Following incubation, cell counts were obtained and infections with virus were initiated. The culture medium was aspirated, and viral inoculum-diluted in medium supplement with 2.5% FBS was added to each well in a final volume of 0.5 mL, achieving a MOI of 0.01 PFU per cell (cells to the virus ratio = 100:1). Infected cells were incubated for 1 h at 37°C, after which the inoculum was removed and replaced with 2 mL of complete media containing 10% FBS. Plates were returned to the incubator for further

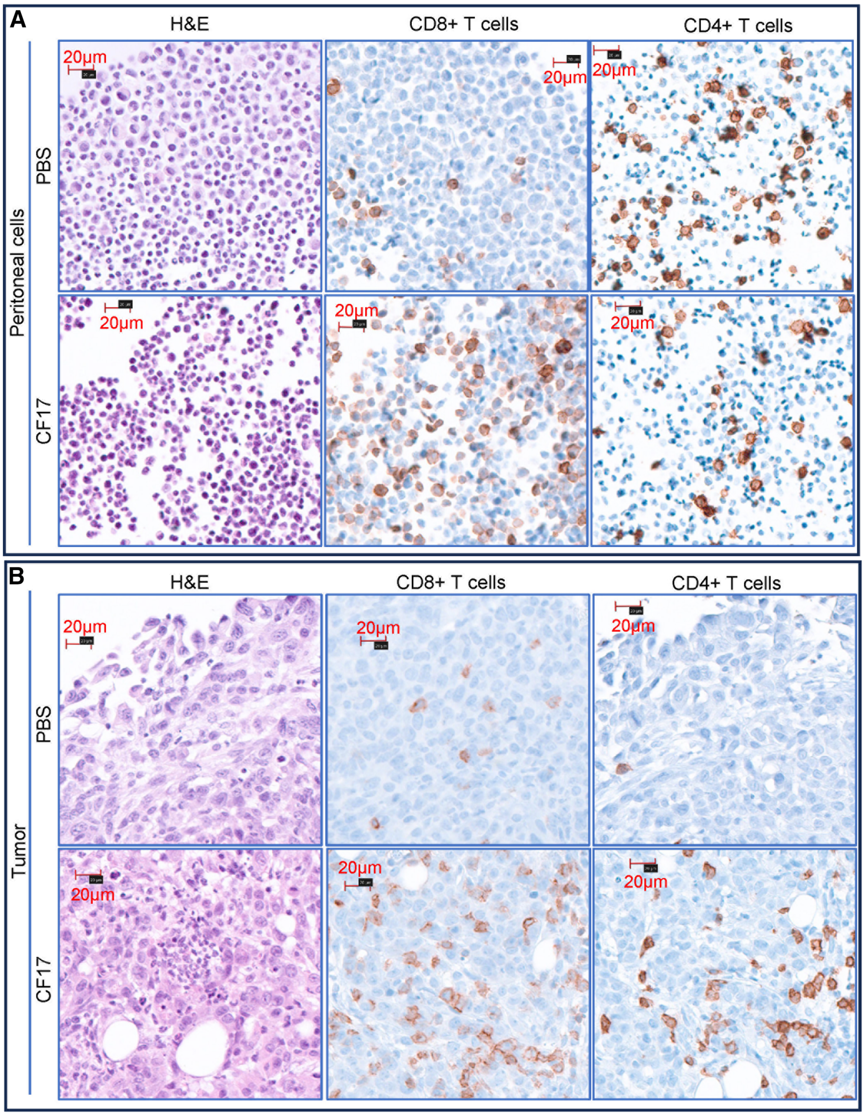

Figure 6. IP CF17 treatment significantly increases CD8⁺ T cells in microenvironment of both peritoneal cavity and peritoneal tumors

(A and B) At day 9 post-IP ACKPY3944 implantation (also on day 2 after third CF17 IP treatment), peritoneal cavity cells (A) and peritoneal tumors (B) were collected and stained with H&E, anti-CD8 antibody, and anti-CD4 antibody using immunohistochemistry. Scale bars, 20 μm.

incubation. At designated time points, cells were harvested by scraping, and viral titer in the lysates was quantified using a standard plaque assay.

Cytotoxicity assay

ACKPY3944 and ACKPY4113 were seeded at 3,000 cells/well in 96-well plates with 100 μL/well of medium supplemented with 10% FBS plus 1% AAS and incubated overnight. The virus thawed on ice and sonicated for one minute. Appropriate MOIs (0.01, 0.1, 1.0, and 10.0) were calculated and prepared for infection in a medium with 2.5% FBS for 20μL/well. Cell viability was measured in triplicate every 24 h for 8 days using MTS cell proliferation assay with CellTiter 96 Aqueous One solution (Promega, Madison, WI) according to manufacture protocol by using a spectrophotometer (Tecan Spark 10M, Mannedorf, Switzerland) at 490 nm.

Establishment of ACKPY3944-ffluc cell line

ACKPY3944 cells were engineered to stably express firefly luciferase (ffluc) by lentiviral transduction using an epHIV7 backbone encoding the ffluc gene under the control of the EF1α promoter, enabling noninvasive bioluminescence imaging (Xenogen). Briefly, ACKPY3944 cells were incubated with polybrene (4 μg/mL; Sigma-Aldrich) and transduced with increasing volumes (1, 5, 10, or 20 μL) of ffluc-expressing lentiviral supernatant. Following transduction and expansion, ffluc expression levels were assessed and compared with those of established murine peritoneal tumor models. The batch exhibiting comparable bioluminescent signal intensity was selected for further expansion and cryopreservation. To minimize potential immunogenic rejection and false-positive tumor regression, a polyclonal population was maintained rather than isolating monoclonal derivatives.

Syngeneic ACKPY3944-ffluc-GCPM mouse model

Animal studies in the syngeneic ACKPY3944-ffluc-GCPM mouse model were performed under the City of Hope Institutional Animal Care and Use Committee (IACUC)-approved protocol (IACUC# 15003). Six-week-old C57BL/6 female and male mice (Charles River, Wilmington, MA) were purchased and acclimatized for 2 weeks. To allow for imaging of peritoneal tumor burden and evaluate the mouse model, the syngeneic mouse model of GCPM was generated by IP injection of 1×10^6 ACKPY3944-ffluc cells in 400 μL PBS into the peritoneal cavity for each mouse (n = 10, 5 male and 5 female mice).

Bioluminescence imaging

To assess peritoneal tumor implantation and progression following IP injection of ACKPY3944-ffluc cells, all animals underwent bioluminescence imaging for luciferase activity. Tumor burden was quantified twice weekly post-implantation. A D-luciferin stock solution (28.5 mg/mL) was prepared by dissolving 1 g of IVISbrite D-Luciferin Potassium Salt Bioluminescent Substrate (PerkinElmer,

catalog#122799-5, Waltham, MA) in 35 mL of phosphate-buffered saline (PBS). Mice received a subcutaneous (SC) injection of 200 μL D-Luciferin per animal and were subsequently imaged using the Lago X optical imaging system (Spectral Instruments Imaging, Tucson, AZ). Bioluminescence signals were analyzed using Aura64 software, and tumor burden was presented as photons/second for regions of interest (photons/sec/ROI).

IP delivery of CF17 *in vivo*

Given the results that 1×10^6 cell implantation demonstrated low deviation of tumor burden (photons/sec/ROI) and stability of tumor growth, the model was chosen for the IP CF17 treatment experiment. In the model, three days after implantation of 1×10^6 ACKPY3944-ffluc cells into the peritoneal cavity, mice were divided into two treatment groups according to the average tumor burden: IP CF17 treatment group (n = 8) and IP PBS control group (n = 8), with extra 3 mice each group for evaluating TME change by harvesting peritoneal lavage and peritoneal tumor. Mice in the IP CF17 treatment group were treated with 3×10^7 pfu CF17 in 400 μL volume of PBS on days 3, 5, and 7 after tumor cell implantation. The mice in the PBS control group received 400 μL PBS on the same dosing schedule. Starting day 7 post-tumor cell implantation, the mice were evaluated weekly to verify IP tumor burden using bioluminescence imaging. Mice were observed and evaluated for tumor burden (luciferase imaging of peritoneal tumor and weight of tumor at death), body weight, and survival. Animals were euthanized if they demonstrated >20% body weight loss, cachexia, or inability to groom and eat, as per institutional guidelines.

Flow cytometric analysis of peritoneal cells

On day 9 after tumor implantation (also on day 2 after third CF17 IP treatment), a total of 6 mice (3 from PBS treatment group and 3 from CF17 treatment group) were sacrificed by exposure to CO_2 overdose. Peritoneal lavage was collected by washing with 5 mL of cold PBS with 2% FBS (2% FBS PBS) using a 10 mL syringe with 25-gauge needle. Cells were counted with hemocytometer, blocked with anti-mouse CD16/32 (BioLegend, catalog# 156604, clone# S17011E), stained with anti-mouse CD45-PerCP/Cyanine5.5 (BioLegend, catalog#103132, clone#30-F11), anti-mouse CD3-FITC (BioLegend, catalog# 100204, clone# 17A2), anti-mouse CD4-APC/Cyanine7 (BioLegend, catalog#100526, clone# RM4-5), and anti-mouse CD8-PE (BioLegend, catalog#100708, clone#53–6.7), and analyzed with a BD LSRFortessa Flow Cytometer and FlowJo software. The numbers of $CD45^+$ leukocytes, cancer cells, $CD3^+$ T cells, $CD4^+$ T cells, and $CD8^+$ T cells in peritoneal lavage were calculated with total cell number (hemocytometer) x percentage of each marker (flow cytometric analysis). Note: cancer cells in peritoneal lavage were defined as $CD45^-$/larger size cells in the dot plot of CD45 versus forward scatter (FSC).

Immunohistochemistry

Peritoneal cells were also stained with H&E, anti-mouse CD8 (catalog# 98941, clone# D4W2Z, Cell Signaling Technology), and anti-mouse CD4 (catalog# ab183685, Clone# EPR19514, Abcam) by using (IHC) performed by Pathology Core of City of Hope. Peritoneal tumors were also harvested on day 9 after tumor implantation (on day 2 after third virus injection), fixed with 10% formalin, embedded in paraffin, and cut into 5-mm-thick sections. Sections were stained with H&E, anti-mouse CD8, or anti-mouse CD4 antibody performed by Pathology Core of City of Hope. Images were obtained using NOP.VIEW2 software.

Statistical analysis

Data are presented as means ± standard error of the mean (SEM). Intergroup comparisons were conducted using either paired or unpaired Student's t test, as appropriate. All statistical tests were two-tailed, with a significance threshold set at p values ≤ 0.05. Survival probabilities were analyzed using Kaplan-Meier curves, and group differences were assessed via the log rank Mantel-Cox test. All statistical computations were performed using GraphPad Prism 8 (GraphPad Software, La Jolla, CA).

DATA AND CODE AVAILABILITY

All data has been presented in the manuscript.

ACKNOWLEDGMENTS

Study in this publication included work performed in the Analytical Cytometry Core, Light Microscopy Digital Imaging Core, Animal Core facility, and Pathology Core facilities supported by the National Cancer Institute of the National Institutes of Health under grant number P30CA033572. The content is solely the responsibility of the authors and does not necessarily represent the official views of the National Institutes of Health. The authors would like to thank Dr. Sandra Ryeom (University of Pennsylvania) for sharing ACKPY3944 and ACKPY4113 cell lines. The authors would like to thank Byungwook Kim, Dr. Maria Hahn, Seonah Kang, Kimberly Acosta, Kasia Rand and Dr. Colin Cook in our laboratory for technical support. The authors would also like to thank Lucy Brown in Analytical Cytometry Core, and Drs. Aimin Li and Zhirong Yin in Pathology Core of City of Hope for supporting the work. Animal studies were performed under the City of Hope Institutional Animal Care and Use Committee (IACUC)-approved protocol. This work was supported by the Department of Surgery Start-Up Grant City of Hope (PI, Yanghee Woo) and the Kuo Family Foundation.

AUTHOR CONTRIBUTIONS

Conception and design, Z.Z., A.Y., and Y.W.; investigation and methodology, Z.Z., A.Y., A.K.P., S.C., S.I.K., J.L., I.M., H.V., and C.C.; analysis and interpretation, Z.Z., A.Y., Y.F., and Y.W.; visualization, Z.Z., S.D., Y.W.; resources and supervision, Y.W. and Y.F.; writing and reviewing, Z.Z., A.Y., S.D., and Y.W.; all authors edited and approved the final manuscript.

DECLARATION OF INTERESTS

Y.F. owns the patent for CF33-OVs and CF17. Y.F., A.P., and S.P. own the patent for CF33-CD19t. CF33-OVs and CF33-CD19t were licensed to Imugene LTD. Y.W. is a member of the scientific advisory board of Imugene LTD.

SUPPLEMENTAL INFORMATION

Supplemental information can be found online at https://doi.org/10.1016/j.omton.2026.201266.

REFERENCES

1. Arnold, M., Abnet, C.C., Neale, R.E., Vignat, J., Giovannucci, E.L., McGlynn, K.A., and Bray, F. (2020). Global Burden of 5 Major Types of Gastrointestinal Cancer. Gastroenterology *159*, 335–349.e15.

2. Sung, H., Ferlay, J., Siegel, R.L., Laversanne, M., Soerjomataram, I., Jemal, A., and Bray, F. (2021). Global Cancer Statistics 2020: GLOBOCAN Estimates of

Incidence and Mortality Worldwide for 36 Cancers in 185 Countries. CA Cancer J. Clin. *71*, 209–249.

3. Zhang, J.F., Lv, L., Zhao, S., Zhou, Q., and Jiang, C.G. (2022). Hyperthermic Intraperitoneal Chemotherapy (HIPEC) Combined with Surgery: A 12-Year Meta-Analysis of this Promising Treatment Strategy for Advanced Gastric Cancer at Different Stages. Ann. Surg Oncol. *29*, 3170–3186.

4. Manzanedo, I., Pereira, F., Rihuete Caro, C., Pérez-Viejo, E., Serrano, Á., Gutiérrez Calvo, A., Regueira, F.M., Casado-Adam, Á., Cascales-Campos, P.A., Arteaga, X., et al. (2019). Cytoreductive Surgery and Hyperthermic Intraperitoneal Chemotherapy (HIPEC) for Gastric Cancer with Peritoneal Carcinomatosis: Multicenter Study of Spanish Group of Peritoneal Oncologic Surgery (GECOP). Ann. Surg Oncol. *26*, 2615–2621.

5. Manzanedo, I., Pereira, F., Pérez-Viejo, E., and Serrano, Á. (2023). Gastric Cancer with Peritoneal Metastases: Current Status and Prospects for Treatment. Cancers (Basel) *15*, 1777.

6. Gamboa, A.C., and Winer, J.H. (2019). Cytoreductive Surgery and Hyperthermic Intraperitoneal Chemotherapy for Gastric Cancer. Cancers (Basel) *11*, 1662.

7. Chia, C.S., You, B., Decullier, E., Vaudoyer, D., Lorimier, G., Abboud, K., Bereder, J.M., Arvieux, C., Boschetti, G., Glehen, O., et al. (2016). Patients with Peritoneal Carcinomatosis from Gastric Cancer Treated with Cytoreductive Surgery and Hyperthermic Intraperitoneal Chemotherapy: Is Cure a Possibility? Ann. Surg Oncol. *23*, 1971–1979.

8. Teixeira Farinha, H., Grass, F., Labgaa, I., Pache, B., Demartines, N., and Hübner, M. (2018). Inflammatory Response and Toxicity After Pressurized IntraPeritoneal Aerosol Chemotherapy. J. Cancer *9*, 13–20.

9. Malfroy, S., Wallet, F., Maucort-Boulch, D., Chardonnal, L., Sens, N., Friggeri, A., Passot, G., Glehen, O., and Piriou, V. (2016). Complications after cytoreductive surgery with hyperthermic intraperitoneal chemotherapy for treatment of peritoneal carcinomatosis: Risk factors for ICU admission and morbidity prognostic score. Surg. Oncol. *25*, 6–15.

10. Lin, D., Shen, Y., and Liang, T. (2023). Oncolytic virotherapy: basic principles, recent advances and future directions. Signal Transduct. Target. Ther. *8*, 156.

11. Chaurasiya, S., Chen, N.G., and Fong, Y. (2018). Oncolytic viruses and immunity. Curr. Opin. Immunol. *51*, 83–90.

12. O'Leary, M.P., Choi, A.H., Kim, S.I., Chaurasiya, S., Lu, J., Park, A.K., Woo, Y., Warner, S.G., Fong, Y., and Chen, N.G. (2018). Novel oncolytic chimeric orthopoxvirus causes regression of pancreatic cancer xenografts and exhibits abscopal effect at a single low dose. J. Transl. Med. *16*, 110.

13. Hammad, M., Cornejo, Y., Flores, L., Hyde, C., Ngai, H.W., Li, M., Dellinger, T.H., Lu, J., Chen, N.G., Mooney, R., et al. (2020). Novel Chimeric Poxvirus CF17 Improves Survival in a Murine Model of Intraperitoneal Ovarian Cancer Metastasis. Mol. Ther. Oncolytics *19*, 278–282.

14. Yang, A., Zhang, Z., Chaurasiya, S., Park, A.K., Lu, J., Kim, S.I., Valencia, H., Fong, Y., and Woo, Y. (2023). Peritoneal-directed chimeric oncolytic virus CF17 prevents malignant ascites and improves survival in gastric cancer peritoneal metastases. Mol. Ther. Oncolytics *31*, 100734.

15. Till, J.E., Yoon, C., Kim, B.J., Roby, K., Addai, P., Jonokuchi, E., Tang, L.H., Yoon, S.S., and Ryeom, S. (2017). Oncogenic KRAS and p53 Loss Drive Gastric Tumorigenesis in Mice That Can Be Attenuated by E-Cadherin Expression. Cancer Res. *77*, 5349–5359.

16. Zhang, Z., Yang, A., Chaurasiya, S., Park, A.K., Kim, S.I., Lu, J., Valencia, H., Fong, Y., and Woo, Y. (2023). Anti-Tumor Immunogenicity of the Oncolytic Virus CF33-hNIS-antiPDL1 against Ex Vivo Peritoneal Cells from Gastric Cancer Patients. Int. J. Mol. Sci. *24*, 14189.

17. Yamamoto, M., Nomura, S., Hosoi, A., Nagaoka, K., Iino, T., Yasuda, T., Saito, T., Matsushita, H., Uchida, E., Seto, Y., et al. (2018). Established gastric cancer cell lines transplantable into C57BL/6 mice show fibroblast growth factor receptor 4 promotion of tumor growth. Cancer Sci. *109*, 1480–1492.

18. Tabuchi, M., Kikuchi, S., Tazawa, H., Okura, T., Ogawa, T., Mitsui, E., Une, Y., Kuroda, S., Sato, H., Noma, K., et al. (2024). Functional remodeling of intraperitoneal macrophages by oncolytic adenovirus restores anti-tumor immunity for peritoneal metastasis of gastric cancer. Mol. Ther. Oncol. *32*, 200806.

19. Blackburn, S.C., and Stanton, M.P. (2014). Anatomy and physiology of the peritoneum. Semin. Pediatr. Surg. *23*, 326–330.

20. Kastelein, A.W., Vos, L.M.C., de Jong, K.H., van Baal, J.O.A.M., Nieuwland, R., van Noorden, C.J.F., Roovers, J.P.W.R., and Lok, C.A.R. (2019). Embryology, anatomy, physiology and pathophysiology of the peritoneum and the peritoneal vasculature. Semin. Cell Dev. Biol. *92*, 27–36.

21. Heise, C., Hermiston, T., Johnson, L., Brooks, G., Sampson-Johannes, A., Williams, A., Hawkins, L., and Kirn, D. (2000). An adenovirus E1A mutant that demonstrates potent and selective systemic anti-tumoral efficacy. Nat. Med. *6*, 1134–1139.

22. Wong, R.J., Joe, J.K., Kim, S.H., Shah, J.P., Horsburgh, B., and Fong, Y. (2002). Oncolytic herpesvirus effectively treats murine squamous cell carcinoma and spreads by natural lymphatics to treat sites of lymphatic metastases. Hum. Gene Ther. *13*, 1213–1223.

23. Haddad, D. (2017). Genetically Engineered Vaccinia Viruses As Agents for Cancer Treatment, Imaging, and Transgene Delivery. Front. Oncol. *7*, 96.

24. Stanford, M.M., and McFadden, G. (2007). Myxoma virus and oncolytic virotherapy: a new biologic weapon in the war against cancer. Expert Opin. Biol. Ther. *7*, 1415–1425.

25. Kim, J.H., Oh, J.Y., Park, B.H., Lee, D.E., Kim, J.S., Park, H.E., Roh, M.S., Je, J.E., Yoon, J.H., Thorne, S.H., et al. (2006). Systemic armed oncolytic and immunologic therapy for cancer with JX-594, a targeted poxvirus expressing GM-CSF. Mol. Ther. *14*, 361–370.

26. Tian, Y., Xie, D., and Yang, L. (2022). Engineering strategies to enhance oncolytic viruses in cancer immunotherapy. Signal Transduct. Target. Ther. *7*, 117.

27. Qiu, Y., Su, M., Liu, L., Tang, Y., Pan, Y., and Sun, J. (2021). Clinical Application of Cytokines in Cancer Immunotherapy. Drug Des. Devel. Ther. *15*, 2269–2287.

28. Pearl, T.M., Markert, J.M., Cassady, K.A., and Ghonime, M.G. (2019). Oncolytic Virus-Based Cytokine Expression to Improve Immune Activity in Brain and Solid Tumors. Mol. Ther. Oncolytics *13*, 14–21.

29. Li, J., O'Malley, M., Urban, J., Sampath, P., Guo, Z.S., Kalinski, P., Thorne, S.H., and Bartlett, D.L. (2011). Chemokine expression from oncolytic vaccinia virus enhances vaccine therapies of cancer. Mol. Ther. *19*, 650–657.

30. Li, F., Sheng, Y., Hou, W., Sampath, P., Byrd, D., Thorne, S., and Zhang, Y. (2020). CCL5-armed oncolytic virus augments CCR5-engineered NK cell infiltration and antitumor efficiency. J. Immunother. Cancer *8*, e000131.

31. Liu, Z., Ravindranathan, R., Li, J., Kalinski, P., Guo, Z.S., and Bartlett, D.L. (2016). CXCL11-Armed oncolytic poxvirus elicits potent antitumor immunity and shows enhanced therapeutic efficacy. Oncoimmunology *5*, e1091554.

32. Moon, E.K., Wang, L.C.S., Bekdache, K., Lynn, R.C., Lo, A., Thorne, S.H., and Albelda, S.M. (2018). Intra-tumoral delivery of CXCL11 via a vaccinia virus, but not by modified T cells, enhances the efficacy of adoptive T cell therapy and vaccines. Oncoimmunology *7*, e1395997.

33. Lee, Y.S., Lee, W.S., Kim, C.W., Lee, S.J., Yang, H., Kong, S.J., Ning, J., Yang, K.M., Kang, B., Kim, W.R., et al. (2020). Oncolytic vaccinia virus reinvigorates peritoneal immunity and cooperates with immune checkpoint inhibitor to suppress peritoneal carcinomatosis in colon cancer. J. Immunother. Cancer *8*, e000857.

34. Bella, Á., Arrizabalaga, L., Di Trani, C.A., Gonzalez-Gomariz, J., Gomar, C., Russo-Cabrera, J.S., Olivera, I., Cirella, A., Fernandez-Sendin, M., Alvarez, M., et al. (2023). Intraperitoneal administration of a modified vaccinia virus Ankara confers single-chain interleukin-12 expression to the omentum and achieves immune-mediated efficacy against peritoneal carcinomatosis. J. Immunother. Cancer *11*, e006702.

35. Wang, M., Luo, Y., Sun, T., Mao, C., Jiang, Y., Yu, X., Li, Z., Xie, T., Wu, F., Yan, H., and Teng, L. (2020). The Ectopic Expression of SurvivinT34A and FilC Can Enhance the Oncolytic Effects of Vaccinia Virus in Murine Gastric Cancer. OncoTargets Ther. *13*, 1011–1025.

36. Liang, M. (2018). Oncorine, the World First Oncolytic Virus Medicine and its Update in China. Curr Cancer Drug Tar *18*, 171–176.

37. Macedo, N., Miller, D.M., Haq, R., and Kaufman, H.L. (2020). Clinical landscape of oncolytic virus research in 2020. J. Immunother. Cancer *8*, e001486.

38. Zeh, H.J., Downs-Canner, S., McCart, J.A., Guo, Z.S., Rao, U.N.M., Ramalingam, L., Thorne, S.H., Jones, H.L., Kalinski, P., Wieckowski, E., et al. (2015). First-in-man

study of western reserve strain oncolytic vaccinia virus: safety, systemic spread, and antitumor activity. Mol. Ther. *23*, 202–214.

39. Manyam, M., Stephens, A.J., Kennard, J.A., LeBlanc, J., Ahmad, S., Kendrick, J.E., and Holloway, R.W. (2021). A phase 1b study of intraperitoneal oncolytic viral immunotherapy in platinum-resistant or refractory ovarian cancer. Gynecol. Oncol. *163*, 481–489.

40. Chaurasiya, S., Chen, N.G., Lu, J., Martin, N., Shen, Y., Kim, S.I., Warner, S.G., Woo, Y., and Fong, Y. (2020). A chimeric poxvirus with J2R (thymidine kinase) deletion shows safety and anti-tumor activity in lung cancer models. Cancer Gene Ther. *27*, 125–135.

41. Shimada, S., Mimata, A., Sekine, M., Mogushi, K., Akiyama, Y., Fukamachi, H., Jonkers, J., Tanaka, H., Eishi, Y., and Yuasa, Y. (2012). Synergistic tumour suppressor activity of E-cadherin and p53 in a conditional mouse model for metastatic diffuse-type gastric cancer. Gut *61*, 344–353.

OMTON, Volume *34*

Supplemental information

Intraperitoneal delivery of the oncolytic virus CF17 improves tumor immunogenicity and survival in gastric cancer peritoneal metastasis

Zhifang Zhang, Annie Yang, Anthony K. Park, Shyambabu Chaurasiya, Sang-In Kim, Jianming Lu, Isabel Monroy, Hannah Valencia, Courtney Chen, Supriya Deshpande, Yuman Fong, and Yanghee Woo

Figure S1

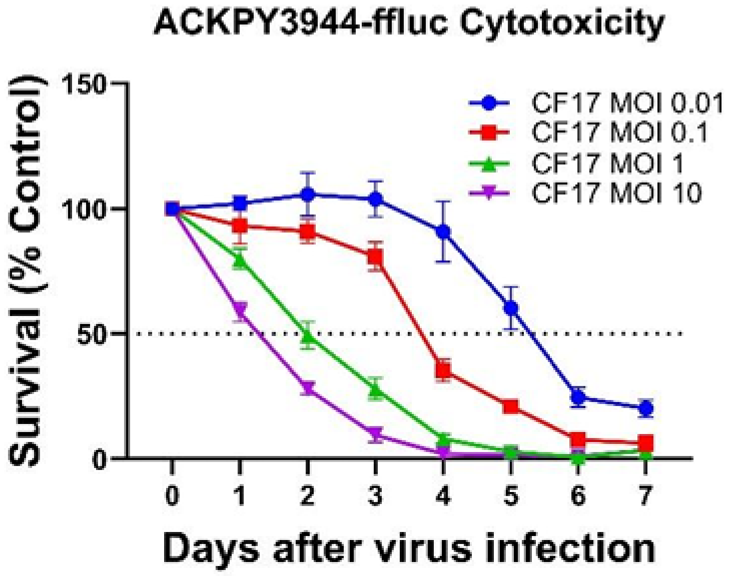

Figure S1. Cytotoxicity of CF17 in ACKPY3944-ffluc cells is similar to that of parental ACKPY3944 cells, related to Figure 2. ACKPY3944 cells were engineered to stably express firefly luciferase (ffluc) via lentiviral transduction and infected with CF17 at the indicated multiplicities of infection (MOIs). Cell survival, relative to mock-infected controls, was measured daily for 7 days post-infection. All experiments were performed thrice, and data are presented as mean ± SEM.

Figure S2

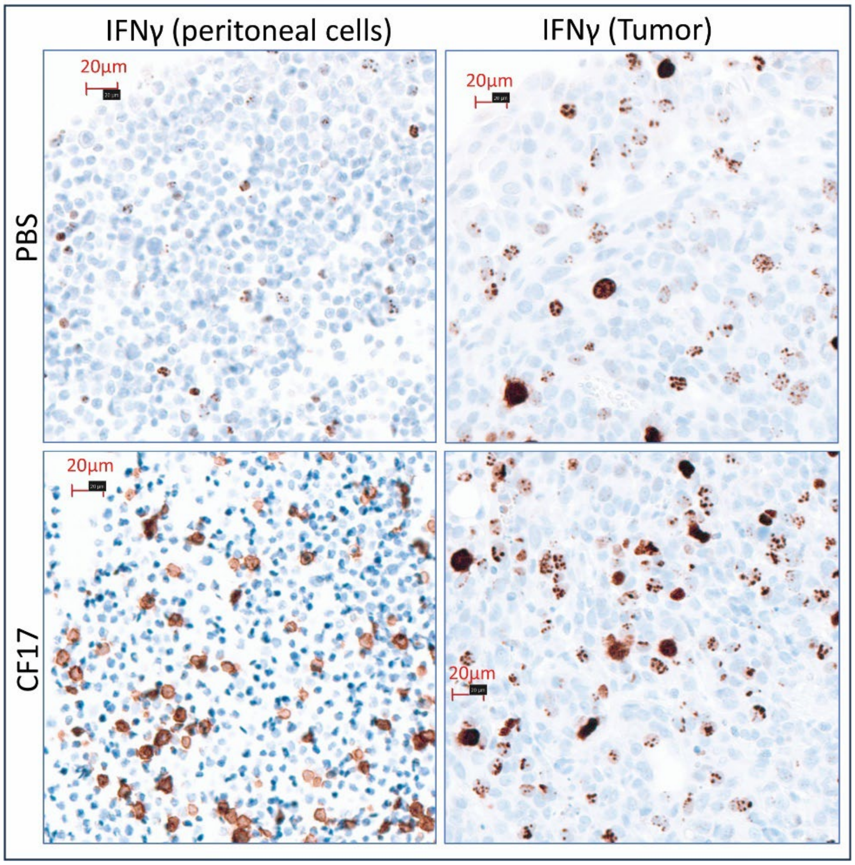

Figure S2. CF17 treatment increases IFNγ⁺ cells in peritoneal cells and tumors after 9 days of treatment, related to Figure 6. Representative immunohistochemistry staining for IFNγ in peritoneal cells (left) and tumor sections (right) from mice treated intraperitoneally with PBS (top) or CF17 (bottom) for 9 days. Scale bar = 20 μm.
